# Public attitudes toward COVID-19 prevention and control in China

**DOI:** 10.3389/fpubh.2024.1292747

**Published:** 2024-05-14

**Authors:** Wei Zhu

**Affiliations:** School of Economics and Management, Changzhou Institute of Technology, Changzhou, Jiangsu, China

**Keywords:** COVID-19, vaccination, pandemic, attitude, China

## Abstract

Based on the data of the Chinese General Social Survey (CGSS) in 2021, this study aims to investigate the public attitudes toward COVID-19 prevention and control in China. The household survey CGSS 2021 contains 8,148 valid samples gathered from 320 communities across 19 provinces in China. The logistic regression model is adopted to examine the relationship between public attitudes and influencing factors. The results show that the vast majority of respondents firmly believe that the government has the authority to implement strict measures; their confidence in the government and in the healthcare system has increased; and they willingly choose to be vaccinated. The regression results suggest that gender, age, health condition, political affiliation, source of information, sense of fairness, socio-economic status, and place of residence are significantly associated with individuals’ attitudes toward COVID-19 prevention and control. These findings highlight the importance for the government to design epidemic or pandemic policies based on data and to tailor them toward specific demographics.

## Introduction

Globally, as of 6 September 2023, there have been 770 million confirmed cases of COVID-19, including 6.9 million deaths, reported to WHO (1). China faced huge challenges in controlling the outbreak of COVID-19 pandemic. To minimize the spread of the pandemic, Chinese government implemented a series of strict prevention and control measures (2). These measures lasted nearly 3 years until the government further optimized its control policies in early December 2022. The spread of COVID-19 pandemic has ended in China, but there has been ongoing debate about China’s response strategies and measures (2–7). These strategies and measures were formulated based on a comprehensive consideration of various factors (2, 3), and among them, people’s attitudes are important ones. Lin et al. (8) found that older age, female gender, and lower Human Development Index were independently associated with greater self-reported compliance with COVID-19 public health measures. Cai and Mason (9) argued that the strong support the Chinese government enjoyed in implementing its COVID-19 control measures emerged from people’s self-interest and nationalistic pride. Yuan et al. (10) showed that institutional trust manipulation increased participants’ willingness to complete the COVID-19 test and that interpersonal trust manipulation increased conscious compliance with prevention norms. Jiang et al. (11) found that marital statuses, attitudes toward government measures, and physical condition were significantly correlated with vaccination willingness. Santos et al. (12) found a significant association between influenza uptake and COVID-19 perceptions. Takamatsu et al. (13) found young age, distrust of the Japanese government’s COVID-19 prevention measures, lack of confidence in vaccine efficacy or safety, low reliance on the vaccine, and belief in COVID-19 conspiracy theories were independently associated with booster vaccine hesitancy. Findings in the literature on the public attitudes toward COVID-19 prevention and control are not conclusive. This study analyzes the public attitudes toward COVID-19 prevention and control based on a large amount of survey data in China, in order to offer insights in response to a possible epidemic or pandemic.

## Methods

### Data source

This study relies on household survey data from the Chinese General Social Survey (CGSS) 2021, conducted by National Survey Research Center, Renmin University of China. Established in 2003, CGSS is the earliest national and continuous academic survey project in China widely utilized by numerous researchers (14, 15). The CGSS employs a stratified three-stage probability proportion to size (PPS) random sample design. The survey of CGSS 2021 was conducted from June 22 to September 30, 2021. It is the latest released CGSS dataset and the first survey of CGSS conducted after the outbreak of COVID-19, and it contains 8,148 valid samples gathered from 320 communities across 19 provinces in China. The questionnaire includes a series of questions about people’s attitudes toward China’s COVID-19 prevention and control.

### Variable definition

There are five dependent variables set based on the questions in CGSS 2021’s questionnaire. First, government’s authority is based on the question: “Do you think the government has the authority to implement the following measures during the severe COVID-19 pandemic period: Shut down businesses or workplaces; Require people to stay at home; Monitor and track infected individuals through digital devices; Require people to wear masks; Prohibit public gatherings; Quarantine infected individuals; Temporarily close primary and secondary schools and kindergartens; Close borders.” Government’s authority is coded as 1 for respondents who choose “Definitely yes” for each measure mentioned above, and 0 for others. Second, confidence in government is coded as 1 for respondents who choose “Increased a lot” or “Increased a bit” to the question: “How the government’s anti-COVID-19 measures have changed your confidence in the government,” and 0 for others. Third, confidence in healthcare system is coded as 1 for respondents who choose “Increased a lot” or “Increased a bit” to the question: “How the government’s anti-COVID-19 measures have changed your confidence in the healthcare system,” and 0 for others. Fourth, vaccination willingness is coded as 1 for respondents who choose “I just want to get vaccinated myself” to the question: “Which of the following statements best fits your situation,” and 0 for others. Fifth, vaccinated is coded as 1 for respondents who choose “Yes” to the question: “Have you been vaccinated against COVID-19,” and 0 for respondents who choose “No.”

According to previous studies (8–11), the independent variables include gender, age, ethnic, education, self-rated health, religion, political affiliation, Internet use, television use, generalized trust, sense of fairness, family size, personal socio-economic status, family economic status, and place of residence. Table 1 presents the descriptive statistics of variables.

**Table 1:** Descriptive statistics.

Variable	Definition	Mean	Standard deviation
Government’s authority	Whether agree that the government has the authority to implement the strict control measures: Definitely yes = 1, Others = 0	0.759	0.428
Confidence in government	Increased = 1, Others = 0	0.883	0.321
Confidence in healthcare system	Increased = 1, Others = 0	0.849	0.358
Vaccination willingness	Willingness to be vaccinated: Yes = 1, No = 0	0.602	0.490
Vaccinated	Have been vaccinated ((at least one dose)): Yes = 1, No = 0	0.734	0.442
Gender	Male = 1, Female = 0	0.452	0.498
Age	Derived by survey year minus respondent’s birth year	51.644	17.574
Ethnic	Ethnic minority = 1 (including all ethnic minorities), Han = 0	0.074	0.261
Education	Years of education: Uneducated = 0, Primary school = 6, Junior high school = 9, Senior high school or technical school = 12, College degree = 15, Bachelor’s degree = 16, Postgraduate or above = 19	9.178	4.623
Self-rated health	Very unhealthy = 1, Relatively unhealthy = 2, Average = 3, Relatively healthy = 4, Very healthy = 5	3.481	1.093
Religion	Adhering to a religion = 1, Not adhering to any religion = 0	0.075	0.263
Political affiliation	Member of the Communist Party of China (CCP): Yes = 1, No = 0	0.119	0.323
Internet use	Internet is used as the primary source of information: Yes = 1, No = 0	0.582	0.493
Television use	Television is used as the primary source of information: Yes = 1, No = 0	0.347	0.476
Generalized trust	Overall, the vast majority of people in this society can be trusted: Strongly disagree = 1, Disagree = 2, Neither agree nor disagree = 3, Agree = 4, Strongly agree = 5	3.638	0.996
Sense of fairness	The general fairness of the current society: Completely unfair = 1, Relatively unfair = 2, Neither fair nor unfair = 3, Relatively fairer = 4, Completely fair = 5	3.454	0.969
Family size	Number of people living together currently	2.289	0.899
Personal socio-economic status	Individual’s socio-economic status: Ordered classification variables, assigned to 1–5	2.598	0.765
Family economic status	Family economic status: Ordered classification variables, assigned to 1–5	3.711	0.899
Place of residence	Urban =1, Rural = 0	0.561	0.496

### Model

Since all five dependent variables are binary, the logistic regression model is adopted to investigate the relationship between a series of influencing factors and public attitudes toward COVID-19 prevention and control. The model is shown in Equation (1):


(1) $$\ln\left(\frac{p}{1-p}\right)=\beta_{0}+\overset{k}{\underset{1}{\sum }}\beta_{j}x_{j}$$

where $p\text{≡}P\left(y=1|\;X\right)$ represents the probability of y=1 , and y ’s include government’s authority, confidence in government, confidence in healthcare system, vaccination willingness, and vaccinated; $\frac{p}{1-p}$ is the odds ratio which represents the ratio of the probability of y=1 to the probability of y=0 ; $x_{j}$ ’s are various independent variables, including gender, age, ethnic, education, etc. (Table 1); $\beta_{0}$ and $\beta_{j}$ ’s are estimated coefficients.

## Analysis

### Government’s authority to implement strict control measures

On average, over 90% of respondents firmly believe that the government has the authority to implement strict measures during the severe COVID-19 pandemic period (Supplementary Table S1).

The regression results for government’s authority are presented in Table 2. Model 1 contains all independent variables, and Model 2 contains only independent variables with a significance level higher than 10% (*p* < 0.100). The odds ratios indicate that gender, age, self-rated health, political affiliation, internet use, sense of fairness, personal socio-economic status, place of residence are statistically significantly correlated to individuals’ attitudes toward COVID-19 prevention and control. The average difference in the odds ratios of these variables between Model 1 and Model 2 is only 1.77%, indicating that the regression results are robust. Individuals who are male, older, in better health conditions, members of CCP, use Internet as the main source of information, possess a higher level sense of fairness, are at a lower personal socio-economic status, and those who reside in urban areas are significantly more likely to agree that the government has the authority to implement strict control measures.

**Table 2:** Regression results for government’s authority.

Variable		Model 1			Model 2	
	OR	*p*	95% CI	OR	*p*	95% CI
Gender	1.197	0.001	1.075–1.332	1.199	0.001	1.079–1.332
Age	1.013	0.000	1.008–1.017	1.013	0.000	1.009–1.017
Ethnic	1.183	0.107	0.965–1.451			
Education	0.997	0.679	0.981–1.012			
Self-rated health	1.085	0.002	1.029–1.143	1.086	0.002	1.031–1.144
Religion	0.869	0.155	0.716–1.055			
Political affiliation	1.197	0.055	0.996–1.437	1.194	0.048	1.001–1.425
Internet use	1.474	0.000	1.190–1.826	1.282	0.000	1.117–1.471
Television use	1.178	0.122	0.957–1.449			
Generalized trust	1.045	0.116	0.989–1.104			
Sense of fairness	1.110	0.000	1.048–1.175	1.128	0.000	1.069–1.191
Family size	1.008	0.596	0.980–1.036			
Personal socio-economic status	0.868	0.000	0.813–0.927	0.864	0.000	0.814–0.918
Family economic status	0.989	0.779	0.913–1.071			
Place of residence	1.324	0.000	1.184–1.480	1.293	0.000	1.163–1.437
Constant	0.625	0.056	0.386–1.012	0.763	0.166	0.520–1.119
Pseudo *R*^2^	0.021			0.014		

OR, Odds Ratio; *p*, *p* value; 95% CI, 95% Confidence Interval. Pseudo *R*^2^ reflects the fit of the model, where a higher value generally indicates a better fit. The same below.

### Changes in confidence due to the control measures

When asked how the government’s anti-COVID-19 measures have changed their confidence in the government and in the health care system, over 60% of respondents indicate that their confidence has increased a lot, and over 20% of respondents indicate that their confidence has increased a bit (Supplementary Table S2; Supplementary Figures S1, S2).

The regression results for confidence in government are presented in the upper part of Table 3. The odds ratios indicate that individuals who are younger, have higher levels of education, are members of CCP, use television as the main source of information, possess a higher level of generalized trust, possess a higher level of sense of fairness, have less family members, are at a higher family economic status, and those who reside in urban areas are significantly more likely to think that the government’s anti-COVID-19 measures have boosted their confidence in the government.

**Table 3:** Regression results for confidence in government and in healthcare system.

Variable		Model 1			Model 2	
	OR	*p*	95% CI	OR	*p*	95% CI
Confidence in government						
Gender	0.903	0.163	0.782–1.042			
Age	0.993	0.021	0.987–0.999	0.990	0.001	0.985–0.996
Ethnic	1.047	0.739	0.801–1.367			
Education	1.032	0.002	1.012–1.052	1.031	0.010	1.012–1.051
Self-rated health	1.053	0.150	0.982–1.129			
Religion	0.945	0.662	0.732–1.219			
Political affiliation	1.299	0.049	1.001–1.687	1.274	0.067	0.983–1.650
Internet use	1.212	0.162	0.926–1.586			
Television use	1.269	0.065	0.985–1.634	1.270	0.062	0.988–1.633
Generalized trust	1.114	0.003	1.038–1.195	1.113	0.003	1.037–1.194
Sense of fairness	1.271	0.000	1.183–1.366	1.267	0.000	1.181–1.361
Family size	0.967	0.061	0.933–1.002	0.967	0.065	0.934–1.002
Personal socio-economic status	0.930	0.113	0.851–1.017			
Family economic status	1.143	0.013	1.029–1.271	1.123	0.013	1.025–1.232
Place of residence	1.301	0.001	1.121–1.510	1.318	0.000	1.140–1.524
Constant	1.485	0.195	0.816–2.703	2.046	0.004	1.249–3.352
Pseudo *R*^2^	0.033			0.031		
Confidence in healthcare system						
Gender	0.899	0.107	0.791–1.023			
Age	0.990	0.000	0.985–0.996	0.990	0.000	0.985–0.995
Ethnic	1.012	0.923	0.797–1.284			
Education	1.026	0.005	1.008–1.045	1.031	0.000	1.015–1.049
Self-rated health	1.051	0.121	0.987–1.119			
Religion	0.868	0.224	0.691–1.090			
Political affiliation	1.110	0.351	0.891–1.383			
Internet use	1.229	0.106	0.957–1.577	1.169	0.065	0.990–1.379
Television use	1.084	0.491	0.861–1.365			
Generalized trust	1.099	0.004	1.031–1.172	1.095	0.005	1.027–1.167
Sense of fairness	1.250	0.000	1.171–1.334	1.251	0.000	1.173–1.334
Family size	0.977	0.142	0.946–1.008			
Personal socio-economic status	0.960	0.323	0.886–1.041			
Family economic status	1.127	0.013	1.025–1.238	1.127	0.005	1.037–1.225
Place of residence	1.095	0.182	0.958–1.251			
Constant	1.633	0.085	0.935–2.854	1.677	0.029	1.055–2.665
Pseudo *R*^2^	0.031			0.029		

The regression results for confidence in healthcare system are presented in the lower part of Table 3. The odds ratios indicate that individuals who are younger, have higher levels of education, use Internet as the main source of information, possess a higher level of generalized trust, possess a higher level of sense of fairness, and those who are at a higher family economic status are significantly more likely to think that the government’s anti-COVID-19 measures have boosted their confidence in the healthcare system.

### Vaccine decision-making

73.42% of respondents report that they have been vaccinated against COVID-19. Among them, 81.95% state that they willingly choose to be vaccinated. The main reasons for being unwilling to get vaccinated are shown in Supplementary Table S3.

The regression results for vaccination willingness (presented in the upper part of Table 4) indicate that individuals who are younger, belong to an ethnic minority, have better health conditions, do not adhere to any religion, are members of CCP, use Internet as the main source of information, possess a higher level of generalized trust, and those who reside in rural areas are significantly more likely to express willingness to get vaccinated.

**Table 4:** Regression results for vaccination willingness and vaccinated.

Variable		Model 1			Model 2	
	OR	*p*	95% CI	OR	*p*	95% CI
Vaccination willingness						
Gender	0.990	0.829	0.899–1.089			
Age	0.984	0.000	0.981–0.988	0.985	0.000	0.982–0.989
Ethnic	1.480	0.000	1.223–1.790	1.497	0.000	1.238–1.809
Education	0.993	0.301	0.980–1.006			
Self-rated health	1.262	0.000	1.205–1.323	1.268	0.000	1.212–1.327
Religion	0.785	0.007	0.659–0.935	0.782	0.006	0.657–0.930
Political affiliation	1.166	0.046	1.002–1.355	1.148	0.058	0.996–1.324
Internet use	1.300	0.007	1.075–1.573	1.206	0.002	1.072–1.358
Television use	1.083	0.392	0.903–1.299			
Generalized trust	1.121	0.000	1.067–1.177	1.128	0.000	1.077–1.182
Sense of fairness	1.025	0.336	0.975–1.078			
Family size	0.996	0.757	0.972–1.021			
Personal socio-economic status	0.969	0.291	0.914–1.027			
Family economic status	1.056	0.128	0.985–1.132			
Place of residence	0.687	0.000	0.621–0.761	0.681	0.000	0.618–0.751
Constant	0.953	0.824	0.623–1.457	1.018	0.918	0.724–1.431
Pseudo *R*^2^	0.045			0.045		
Vaccinated						
Gender	0.826	0.001	0.741–0.922	0.838	0.001	0.753–0.933
Age	0.970	0.000	0.965–0.975	0.968	0.000	0.964–0.973
Ethnic	1.426	0.002	1.138–1.787	1.417	0.002	1.131–1.776
Education	1.012	0.110	0.997–1.027			
Self-rated health	1.300	0.000	1.234–1.370	1.299	0.000	1.234–1.367
Religion	0.781	0.011	0.645–0.946	0.780	0.011	0.644–0.943
Political affiliation	1.257	0.007	1.064–1.484	1.291	0.002	1.098–1.517
Internet use	1.455	0.000	1.192–1.777	1.460	0.000	1.286–1.657
Television use	1.018	0.855	0.843–1.228			
Generalized trust	1.076	0.011	1.017–1.138	1.070	0.012	1.015–1.129
Sense of fairness	0.979	0.460	0.924–1.036			
Family size	1.037	0.011	1.008–1.067	1.036	0.015	1.007–1.065
Personal socio-economic status	0.941	0.072	0.881–1.005	0.935	0.026	0.881–0.992
Family economic status	0.979	0.602	0.905–1.060			
Place of residence	0.623	0.000	0.554–0.700	0.637	0.000	0.570–0.713
Constant	5.486	0.000	3.437–8.757	5.901	0.000	3.914–8.897
Pseudo *R*^2^	0.104			0.103		

The regression results for vaccinated (presented in the lower part of Table 4) suggest that individuals who are female, younger, belong to an ethnic minority, have better health conditions, do not adhere to any religion, are members of CCP, use Internet as the main source of information, possess a higher level of generalized trust, have more family members, are at a lower personal socio-economic status, and those who reside in rural areas are significantly more likely to be vaccinated.

## Discussion

This study utilizes a dataset from a large-scale household survey, comprising a wide range of Chinese adults who are surveyed about their attitudes toward China’s COVID-19 prevention and control. The results demonstrate that: the vast majority of respondents firmly believe that the government has the authority to implement strict measures during severe COVID-19 pandemic period; their confidence in the government and in the healthcare system has increased; and they willingly choose to be vaccinated. These findings reveal a high level of public compliance with strict anti-COVID-19 measures in China, which is in line with prior research (9, 11). Furthermore, the results suggest that gender, age, health condition, political affiliation, source of information, sense of fairness, socio-economic status, and place of residence are significantly associated with individuals’ attitudes toward China’s COVID-19 prevention and control. On the one hand, sociodemographic characteristics will affect their attitudes toward the government’s policies (9, 11); on the other hand, the effects of pandemic prevention and control measures vary across socioeconomic groups (16). These findings highlight the importance for the government to design epidemic or pandemic policies based on data and to tailor them toward specific demographics (17). Although the majority of the respondents strongly supported the government’s policies against COVID-19 during the first 2 years of the pandemic, people’s attitudes will change when the situation changes or the information they have got changes (9), especially among young people (13). Therefore, the government should pay more attention to the changes in public attitudes, provide clear and trustworthy information (17, 18), send personalized messages (especially targeted and tailored messages for the young generation) (13), and then make policy adjustments in due time.

## Conclusion

The findings of this study support previous studies that reveal a high level of public compliance with strict anti-COVID-19 measures in China. Furthermore, it underlines the impact of gender, age, health condition, political affiliation, source of information, sense of fairness, socio-economic status, and place of residence on individuals’ attitudes toward COVID-19 prevention and control. These findings highlight the importance for the government to design epidemic or pandemic policies based on specific demographics data and to make timely policy adjustments in response to changes in people’s attitudes.

## Data availability statement

Publicly available datasets were analyzed in this study. This data can be found here: http://www.cnsda.org/index.php.

## Author contributions

WZ: Data curation, Formal analysis, Writing – original draft, Writing – review & editing.
