# Supplementary material for: Public attitudes toward COVID-19 prevention and control in China
Source: Front Public Health. 2024 May 14;12:1292747. doi: 10.3389/fpubh.2024.1292747 (PMC11130392; doi:10.3389/fpubh.2024.1292747)
Supplement: Supplementary file 1 [file Data_Sheet_1.docx]

**Supplemental file**

**TABLE S1** Attitudes on whether the government has the authority to implement strict control measures.

| **Control measures** | **Definitely yes** | **Probably yes** | **Probably no** | **Definitely**  **no** | **Unable to select** | **Refuse to answer** |
| --- | --- | --- | --- | --- | --- | --- |
| Shut down businesses or workplaces | 87.46% | 8.35% | 1.24% | 1.15% | 1.76% | 0.05% |
| Require people to stay at home | 90.75% | 6.57% | 1.04% | 0.85% | 0.76% | 0.04% |
| Monitor and track infected individuals through digital devices (such as mobile phones) | 89.09% | 6.91% | 1.23% | 1.13% | 1.61% | 0.04% |
| Require people to wear masks | 96.02% | 3.23% | 0.14% | 0.17% | 0.42% | 0.02% |
| Prohibit public gatherings | 94.23% | 4.09% | 0.43% | 0.60% | 0.61% | 0.04% |
| Quarantine infected individuals | 96.80% | 2.33% | 0.18% | 0.17% | 0.49% | 0.02% |
| Temporarily close primary and secondary schools and kindergartens | 93.45% | 4.52% | 0.83% | 0.23% | 0.93% | 0.04% |
| Close borders | 93.00% | 4.12% | 0.63% | 0.39% | 1.80% | 0.05% |

**TABLE S2** Perception of the changes in confidence due to the control measures

| **Changes in confidence** | **Increased a lot** | **Increased a bit** | **Basically unchanged** | **Decreased a bit** | **Decreased a lot** | **Unable to select** | **Refuse to answer** |
| --- | --- | --- | --- | --- | --- | --- | --- |
| Confidence in government | 66.91% | 21.43% | 9.11% | 0.45% | 0.36% | 1.60% | 0.15% |
| Confidence in healthcare system | 60.13% | 24.78% | 11.25% | 0.59% | 0.69% | 2.45% | 0.11% |

FIGURE S1 Changes in confidence. in the government (n,%). FIGURE S2 Changes in confidence. in the health care system (n,%).

**TABLE S3** Reasons mentioned by the respondents for being unwilling to get vaccinated against COVID-19

| **Reasons** | **Frequency** | **Percentage** |
| --- | --- | --- |
| Not meeting the vaccination requirements | 1,388 | 43.11% |
| Concerns about adverse health effects after vaccination | 849 | 26.37% |
| Considering vaccination too troublesome | 300 | 9.32% |
| Believing that there is no need to get vaccinated due to the well-controlled epidemic situation in China | 285 | 8.85% |
| Doubts about the effectiveness of the vaccine in preventing the disease | 207 | 6.43% |
| Not knowing where to get vaccinated | 121 | 3.76% |
| Feeling that there is no need to get vaccinated since others have already done so | 85 | 2.64% |
| Concerns about the government’s inadequate management of vaccines (such as fake vaccines, ineffective vaccines, etc.) | 49 | 1.52% |
| Wanting vaccines from other countries | 2 | 0.06% |
| Other reasons | 600 | 18.63% |
| Refuse to answer | 9 | 0.28% |
